# Estimation of Molecular Pairwise Relatedness in Autopolyploid Crops

**DOI:** 10.1534/g3.120.401669

**Published:** 2020-10-13

**Authors:** Rodrigo R. Amadeu, Leticia A. C. Lara, Patricio Munoz, Antonio A. F. Garcia

**Affiliations:** * Horticultural Sciences Department, University of Florida, Gainesville, FL; † Department of Genetics, Luiz de Queiroz College of Agriculture, University of São Paulo, Brazil; ‡ The Roslin Institute and Royal (Dick) School of Veterinary Studies, The University of Edinburgh, Edinburgh, UK

**Keywords:** Relationship Polyploid Autotetraploid Mendelian sampling variance Molecular marker SNP

## Abstract

A suitable pairwise relatedness estimation is key to genetic studies. Several methods are proposed to compute relatedness in autopolyploids based on molecular data. However, unlike diploids, autopolyploids still need further studies considering scenarios with many linked molecular markers with known dosage. In this study, we provide guidelines for plant geneticists and breeders to access trustworthy pairwise relatedness estimates. To this end, we simulated populations considering different ploidy levels, meiotic pairings patterns, number of loci and alleles, and inbreeding levels. Analysis were performed to access the accuracy of distinct methods and to demonstrate the usefulness of molecular marker in practical situations. Overall, our results suggest that at least 100 effective biallelic molecular markers are required to have good pairwise relatedness estimation if methods based on correlation is used. For this number of loci, current methods based on multiallelic markers show lower performance than biallelic ones. To estimate relatedness in cases of inbreeding or close relationships (as parent-offspring, full-sibs, or half-sibs) is more challenging. Methods to estimate pairwise relatedness based on molecular markers, for different ploidy levels or pedigrees were implemented in the AGHmatrix R package.

Pairwise relatedness (*r*) estimation is a central point in population and quantitative genetics studies, being used for distinct applications. For example, the estimation of genetic variance components is a function of *r* (Lynch *et al.* 1998). Based on the variance components estimation, it is possible to predict breeding values (Henderson 1976) and to perform genomic selection (VanRaden 2008). This value can also be used to correct for kinship and population structure in genome-wide association studies (GWAS) (Korte and Farlow 2013). *r* is a component to plan and optimize crosses for conservation or breeding programs (Gorjanc *et al.* 2018). In conservation genetics, it is used to design crosses that avoid inbreeding enhancing genetic variability (Lynch and Ritland 1999). In breeding, crosses are planned to combine parents with distinct genetic backgrounds, enhancing heterosis and accelerating the development of improved cultivars (Wricke and Weber 1986). Despite the usage of *r* estimates based on molecular data on polyploid crops as blueberry (Ferrão *et al.* 2018; de Bem Oliveira *et al.* 2019), potato (Endelman *et al.* 2018; Amadeu *et al.* 2020), and different forages (de C. Lara *et al.* 2019; Matias *et al.* 2019), there is a lack of studies about the computation of pairwise relatedness in polyploidy species where researchers need to be able to estimate *r* in the best possible way.

Polyploidy is considered a major evolutionary force in plants (Soltis *et al.* 2014) and is presented in agricultural crops. Such force, also called whole genome duplication, generally results in instant speciation, and it is driven by autopolyploidy and allopolyploidy. The association of genomes from different species into one is called allopolyploidy, which is an interspecific hybridization followed by a chromosome doubling, or vice-versa (Gallais 2003). An autopolyploidy, on the other hand, involves a *per se* chromosome doubling through the association of two unreduced gametes. Unlike allopolyploids, meiotic pairing in autopolyploids can involve formation of multivalent structures caused by the pairing between more than two homologous chromosomes leading to the term known as polysomic inheritance (Gallais 2003; Mackay *et al.* 2019). Although the division into allo- and autopolyploids is convenient, it is rare to find species that present pure allopolyploid or autopolyploid segregation (Soltis *et al.* 2014). As pointed by Doyle and Sherman-Broyles (2017), it is botanically and genetically troublesome to define a given species as autopolyploid or allopolyploid. The majority of polyploid species is found in a gray area between having a complete dissomic (allopolyploidy) or polyssomic (autopolyploidy) inheritance. In this work, by convenience, we split agricultural crops into allopolyploids and autopolyploids, meaning that they are mostly known as one type or another and their meiosis mainly follows its pertinent classification. However, there is no way to confirm they are exclusively of that type. Following such statement, distinct and important crops, such as forages, potatoes, blueberries, strawberry, and sugarcane are commonly classified as autopolyploids (Table 1). Among allopolyploids, wheat is one of the most studied species classified as such.

**Table 1:** Example of autopolyploid crops

Common name^a^	Species	Cytotype	Reference
alfalfa	*Medicago sativa*	2n = 4x	Gallais (2003)
blueberry	*Vaccinium* spp.	2n = 2x to 6x	Boches *et al.* (2006)
brachiaria grass	*Brachiaria* spp.	2n = 2x, 4x	Penteado *et al.* (2000)
chrysanthemums	*Chrysanthemum* spp.	2n = 2x to 10x	Wang *et al.* (2015)
guinea grass	*Panicum maximum*	2n = 2x to 8x	Savidan (1980)
leek	*Allium porrum*	2n = 4x	Gallais (2003)
potato	*Solanum tuberosum*	2n = 4x	Gallais (2003)
rose	*Rosa* spp.	2n = 2x to 10x	Saint-Oyant *et al.* (2018)
strawberryb	*Fragaria* x *ananassa*	2n = 4x	Hirakawa *et al.* (2014)
sugarcane^c^	*Saccharum officinarum*	2n = 10x	D’Hont *et al.* (1998)
sweet potato	*Ipomoea batatas*	2n = 6x	Gallais (2003)
switchgrass	*Panicum virgatum*	2n = 4x, 8x	Lipka *et al.* (2014)
tea	*Camellia sinensis*	2n = 4x	Gallais (2003)
yam	*Dioscorea alata*	2n = 6x	Gallais (2003)

a This is not an exhaustive list: the cytotypes and crops are not strict to this table and we do not incorporate allopolyploidy events.

b Strawberry is an allopolyploid with autopolyploid events.

c Modern sugarcane cultivars are typically interspecific hibrids between autopolyploid *Saccharum officinarum*, *Saccharum spontaneum*, and other *Saccharum* species with varied ploidy level.

Motivated by the importance to obtain reliable estimates of relatedness in autopolyploids, the objective of this study was to investigate different statistical approaches to compute pairwise relatedness. While statistical methods for it are relatively mature for diploid analyses, they remain somewhat under-explored in the polyploid literature, and to our knowledge there are no clear guidance about how to use them in such scenario. Here, through simulations based on real pedigree data, we surveyed autopolyploid Mendelian sampling variance and compared different statistical approaches to compute pairwise relatedness. We select eight different methods as the most relevant ones, as will be presented in the following section.

## Theory

The computation of pairwise relatedness based on a given genealogy is part of classic studies described by Wright (1922). In diploids, Wright’s coefficient of relationship is defined as the probability that a random allele of a given genotype is identical-by-descent to a random allele taken from another genotype. The additive covariance *A* among two individuals *X* and *Y* can be expressed as $\sigma_{XY}=r_{XY}\sigma_{A}^{2}$, where $\sigma_{A}^{2}$ is the additive genetic variance (Lynch and Ritland 1999; Lynch *et al.* 1998). In allopolyploid species, meiosis generally behaves as having disomic inheritance (Luo *et al.* 2006) and, therefore, a diploid framework can be straightforward extended to allopolyploid analyses. However, the same is not valid for autopolyploids, since its meiosis could involve polysomic inheritance. In this case, the coefficient of relationship between individuals *X* and *Y* is given by $r_{XY}=2v\theta_{XY}$ where $\theta_{XY}$ is the coefficient of kinship and *v* is the species’ gametic ploidy level (*e.g.*, if autohexaploid, 2n=6x, v=3) (Kerr *et al.* 2012). Although the terms of the covariance for the effects due to allelic interaction among two individuals *X* and *Y* expand as the ploidy level increases (with digenic, trigenic, quadrigenic, and so on), the additive covariance between two individuals is the same as in diploids: $\sigma_{XY}=r_{XY}\sigma_{A}^{2}$ (Kempthorne 1957). The pairwise relatedness *r* for a given locus can then be generalized to:

$$r_{XY}=\sum_{i=0}^{2v}\frac{i{\text{Δ}}_{i}}{2v}$$ (1)

where ${\text{Δ}}_{i}$ is the probability to have a set of *i* allele(s) identical-by-descent between two individuals *X* and *Y* for this locus (Gallais 2003; Huang *et al.* 2015).

### Autopolyploid identical-by-descent pairwise relatedness

A general algorithm to compute identical-by-descent pairwise relatedness was proposed for autopolyploid species by Kerr *et al.* (2012). This algorithm is similar to the one derived by Henderson (1976) for diploids. It is a recursive algorithm, where recursiveness is given by the fact that the relatedness between individuals *X* and *Y* is half of the summation of the relatedness between *X* and the parents of *Y*. Considering ploidy levels, in Kerr *et al.* (2012), the inbreeding (relatedness of the individual with itself) is computed including double-reduction fraction and the chance to inherit sets of alleles identical-by-descent from the parents. Double-reduction occurs when one gamete receives two segments of the same homolog because of the multivalent pairing (Gallais 2003). This cytogenetic phenomenon increases the overall inbreeding in the population and it has been well studied in potato (Bourke *et al.* 2015) and yeast (Stift *et al.* 2010). The chance to inherit sets of alleles identical-by-descent is not possible in diploids, where only one allele from each parent is transmitted to the descendants. As consequence, dominance effects in diploid are not inherited. In contrasts, sets of alleles can be passed from parent to offspring under autopolyploid meiosis. This can be translated into inbreeding inheritance from one generation to the next which might result in a buffering effect to decrease inbreeding in autopolyploid populations.

### Autopolyploid identical-by-state pairwise relatedness

Pairwise relatedness can also be estimated with molecular markers based on alleles identity-by-state. A lack of pedigree records is common in many breeding populations or natural populations. Consequently, the estimation of inbreeding and pairwise relatedness often can only be performed through the use of molecular markers. In diploids, based on molecular markers, Lynch and Ritland (1999) described different pairwise relatedness estimators for multiallelic loci. Using biallelic markers (as single nucleotide polymorphisms - SNPs), VanRaden (2008) and Yang *et al.* (2010) proposed pairwise relatedness estimators. On the other hand in autopolyploidy and considering multiallelic loci, Huang *et al.* (2014, 2015) presented, respectively, a method-of-moments (MM) and a maximum-likelihood (ML) molecular pairwise relatedness estimators. The same authors implemented these multiallelic methods in the PolyRelatedness software, additionally with three others extended methods for polyploids, algorithms based on Loiselle *et al.* (1995) (LO), Ritland (1996) (RI), and Weir (1996) (WE). With biallelic markers, there is an extension of VanRaden (2008) coefficient considering polyploid dosage (VR) (adapted from Ashraf *et al.* (2016)) and two methods to estimate relatedness proposed by Slater *et al.* (2016): pseudo-diploid (PD) and full-autopolyploid (FA). The main differences across the aforementioned methods are related to how each allele is weighted, and whether it is or not corrected by the allele frequency. RI, LO, and WE are methods based on similarity index of each allele in a given locus. MM and ML are based on the estimation of higher order coefficients (Δ) that are later combined to estimate *r* (Equation 1). VR and PD are a simple correlation between the loci vectors of the individuals. VR considers multiple dosage and PD just diploid dosage (all heterozygous are merged into one class, with “hidden heterozygotes”). FA considers the correlation between individuals with the same genotype with no explicit additive model between different dosages (*i.e.*, additive and non-additive effects are confounded).

### identical-by-descent and identical-by-state correlation

The correlation between pairwise relatedness based on identical-by-descent and on identical-by-state is not necesssarily of high magnitude. In a real scenario - with linkage and finite number of loci - the Mendelian sampling variance and the allelic frequencies can affect relatedness estimation. Mendelian sampling is the level of relatedness variability due to gametic sampling and recombination coming from the parents (Isik *et al.* 2017). As an example, consider one locus and two full-sib individuals derived from a cross between two parents with given genotypes AB and CD. Here, for a given locus, one sibling may be AC and the other be BD, and therefore being genotypically unrelated for this locus (r=0). Another possible result would be when both siblings received the same set of alleles, resulting in r=1. Although the expected values are the same when identical-by-descent and identical-by-state methods are applied, the identical-by-state methods are able to capture the deviation due to Mendelian sampling. Given genomic length and number of chromosomes, each relationship case has a different Mendelian sampling standard deviation. In full-sibs, for instance, Hill and Weir (2011) found that the standard deviation of *r* in humans is 0.0392. The same authors also showed that variation increases with the expected (based on the pedigree) *r*. To illustrate, the standard deviation of *r* for full-sibs is higher than the standard deviation between two cousins. Additionally, population allelic frequencies can change the pairwise relatedness between a given pair of individuals. Two individuals may not have any alleles identical-by-descent, however, these alleles may be, by chance, identical-by-state. This can increase relatedness between individuals. Due to sampling, two unrelated individuals have a chance to be genotypically similar in a given locus and, consequently, to show high *r* value when estimated based on markers.

## Materials And Methods

### Pedigree

**Figure 1:**
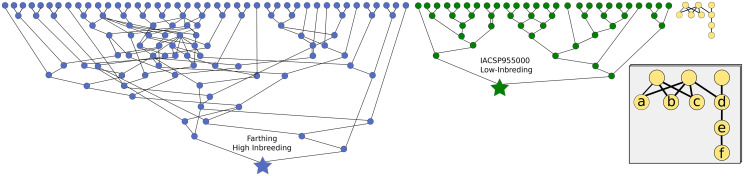
Genealogies from blueberry cultivar *Farthing* (high-inbreeding, in blue), sugarcane cultivar *IACSP955000* (low-inbreeding, in green), and common relationships (in yellow). Circles represent genotypes. Ancestrals genotypes on top row. Stars represents the cultivars. Lines represents gametic transmission. In detail, relationship examples as full-sibs (between *a* and *b*), half-sibs (*b* and *c*), parent-offspring (*d* and *e*), uncle-nephew (*c* and *e*), grandparent-grandoffspring (*d* and *f*), and granduncle-grandnephew (*c* and *f*).

To evaluate the influence of relationship levels in the estimation, we considered three different pedigree scenarios, two pedigrees based on two autopolyploid commercial varieties contrasting for inbreeding level (sugarcane and blueberry) and one pedigree with common relatedness. The first pedigree (high-inbreeding) is derived from *Farthing* Lyrene (2008) which is an autotetraploid blueberry commercial variety, released in 2007, from the Blueberry Breeding Program of the University of Florida, USA. This pedigree comprises a total of 77 lines of records, with a high number of inbreeding events, hereafter called as high-inbreeding genealogy. At the end, we built a pedigree with 183 lines, being 73 ancestral genotypes. The second pedigree (low-inbreeding) is the one of the *IACSP955000* (Portal do Governo do Estado de São Paulo 2007) which is a sugarcane commercial variety, a complex autopolyploid, released in 2007, from the Sugarcane Breeding Program of the Agronomic Institute of Campinas, Brazil. This pedigree comprises a total of 27 lines of records, with a low number of inbreeding events, hereafter called as low-inbreeding genealogy. The third pedigree accounted for common relatednesses, we considered relationships usually used in genetical studies (Figure 1): parent-offspring, grandparent-grandoffspring, full-sibs, half-sibs, uncle-nephew, and granduncle-grandnephew. Unrelated relationships were considered as check. The expected inbreeding and relatedness between individuals were computed following Kerr *et al.* (2012) and implemented in the AGHmatrix software v2.0 (Amadeu *et al.* 2016).

### Simulations

Based on the designed pedigree (Figure 1), we simulated the genotypes using the methodology implemented in the PedigreeSim software V2.0 (Voorrips and Maliepaard 2012). We considered seven combinations of ploidy and meiosis (allowing or not allowing formation of quadrivalent), as follows: i) diploids, ii) autotetraploids with only bivalent pairing, iii) autotetraploids allowing for quadrivalent pairing, iv) autohexaploids with only bivalent pairing, v) autohexaploids allowing for quadrivalent pairing, vi) autooctaploids with only bivalent pairing, and vii) autooctaploids allowing for quadrivalent pairing. The scenarios with quadrivalent pairing allow formation of quadrivalents with expected probabilities as 2/3 of the autotetraploid meiosis, 9/10 of the autohexaploid meiosis, and 24/25 of the autooctaploid meiosis. This is set as with the arguments ”NATURAL PAIRING = 1” in PedigreeSim software. Quadrivalent formation has as consequence a small fraction of double-reduction. Noteworthy that those proportions are expected values assuming random assortment of the chromosome ends and more realistic fractions would depend on the biological model and a deep understanding of its meiotic process which is rarely available. No preferential pairing was simulated. For each combination of ploidy and meiosis, we performed 100 independent simulations. The simulated genome consisted of 10 chromosomes, each one with 100 centiMorgans and one locus every 0.1 cM, - summing up 10,000 loci. All the ancestral genotypes were assumed as unrelated and with unique alleles.

### Realized pairwise relatedness

Genetic phenomena related to Mendelian sampling, polysomic inheritance, linkage, and numbers of loci and chromosomes can result in differences between simulated and expected relatedness. Therefore, for each simulated population, we computed the observed (realized) coefficient of relatedness ($\hat{r}$):

$${\hat{r}}_{XY_{obs}}={\hat{r}}_{obs}=\frac{1}{L}\sum_{j=1}^{L}\sum_{i=0}^{2v}\frac{i{\text{Δ}}_{i\left(j\right)}}{2v}$$ (2)

During the simulation, it is possible to track the origin of all alleles. Hence, considering all simulated alleles, the observed probability of an allele to be identical-by-state is the same of an allele to be identical-by-descent. Knowing beforehand the genotypes, we computed the ${\text{Δ}}_{i}$ for each pair of genotypes within each locus *j*. In this procedure, the parameter space for each observed ${\text{Δ}}_{i}$ is 0 or 1, *i.e.*, the individuals *X* and *Y* share *i* alleles within the locus, or they do not. Then, those values were averaged across all *L* loci.

### Locus and allele sampling scenarios

From the seven combination of ploidy and meiosis simulated populations, we evaluated 952 distinct scenarios with 100 repetitions each. These come from a combination between number of loci, number of alleles, and allele probability distribution. The numbers of loci sampled were 5, 10, 15, 20, 50, 100, 500, and 1,000. The number of alleles sampled were 2, 3, 4, 5, 10, 15, and 20. The allelic distributions were uniform (1:1:1: …) and triangular (1:2:3: …). For the biallelic scenarios, we also sampled the alleles from binomial distributions with probability to sampling the alleles following the ratios 1:3, 1:4, and 1:9. The allele sampling was applied to the ancestral alleles and, then, all the population (progeny) was later recorded following this sampling. As an example, a tested scenario is the autohexaploid with multivalent pairing, with 500 loci, each locus with 2 alleles where the ancestral alleles were sampled from a binomial distribution 1:4. At the end, we obtained a total of 952 scenarios with 100 sampling population each (392 from uniform distribution, 392 from triangular distribution, 56 biallelic with 1:3 binomial distribution, 56 with biallelic 1:4 binomial distribution, and 56 biallelic with 1:9 binomial distribution). All the scenarios are described in Supplementary Data S1.

### Pairwise genomic relationship estimation

We considered eight methods to estimate the pairwise genomic relationship (Table 2): five multiallelic (LO, RI, WE, MM, and ML) and three biallelic (VR, PD, and FA). For simplicity in notation, we will remove the XY subscript, hereafter ${\hat{r}}_{XY_{method}}={\hat{r}}_{method}$.

**Table 2:** Methods evaluated

Abbreviation	Marker data*^a^*	Description*^b^*	References
LO	Multiallelic	Extended method-of-moments	Loiselle *et al.* (1995); Huang *et al.* (2014)
RI	Multiallelic	Extended method-of-moments	Ritland (1996); Huang *et al.* (2014)
WE	Multiallelic	Extended method-of-moments	Weir (1996); Huang *et al.* (2014)
MM	Multiallelic	Method-of-moments	Huang *et al.* (2014)
ML	Multiallelic	Maximum-likelihood estimator	Huang *et al.* (2015)
VR	Biallelic	Extended relationship matrix	VanRaden (2008); Ashraf *et al.* (2016)
PD	Biallelic	Extended pseudo-diploid relationship matrix	Yang *et al.* (2010); Slater *et al.* (2016)
FA	Biallelic	Full-autopolyploid relationship matrix	Slater *et al.* (2016)

a Multiallelic methods are also biallelic methods.

b Extended methods refer to methodologies originally proposed by the first reference and later extended for autopolyploids in the second reference.

#### LO:

It is a method for autopolyploidy based on Loiselle *et al.* (1995) and extended for autopolyploidy by Huang *et al.* (2014):

$${\hat{r}}_{LO}=2v\frac{\sum_{j=1}^{L}\sum_{i=1}^{k_{j}}\left[\left(S_{ijx}-p_{ij}\right)\left(S_{ijy}-p_{ij}\right)\right]}{\sum_{j=1}^{L}\sum_{i=1}^{k_{j}}\left[\left(p_{ij}\right)\left(1-p_{ij}\right)\right]}$$ (3)

*L* is the total number of loci, *j* is the current locus, $k_{j}$ is the number of alleles of the current locus *j*, $S_{ijx}$ and $S_{ijy}$ are the similarity coefficients of the alleles *i* of locus *j* (this similarity is the frequency of the allele *i* in the genotype), $p_{ij}$ is the frequency of the allele *i* of the locus *j* in the population, *v* is the species’ gametic ploidy level.

### RI

It is a method for autopolyploidy based on Ritland (1996) and extended by Hardy and Vekemans (2002) and Huang *et al.* (2014):

$${\hat{\theta }}_{XY}=2v\frac{\sum_{j=1}^{L}\left[\sum_{i=1}^{k_{j}}\left(S_{ijx}S_{ijy}/p_{ij}\right)-1\right]}{\sum_{j=1}^{L}\left(k_{j}-1\right)}$$ (4)

$${\hat{r}}_{RI}=\frac{1}{2}\;{\hat{\theta }}_{XY}\left(\frac{1}{{\hat{\theta }}_{XX}}+\frac{1}{{\hat{\theta }}_{YY}}\right)$$ (5)

#### WE:

It is a method for autopolyploidy based on Li and Horvitz (1953) and Weir (1996), extended by Huang *et al.* (2014):

$${\hat{r}}_{WE}=2v\frac{\sum_{j=1}^{L}[\sum_{i=1}^{k_{j}}\left(S_{ijx}S_{ijy}-p_{ij}^{2}\right)}{L-\sum_{j=1}^{L}\sum_{i=1}^{k_{j}}p_{ij}^{2}}$$ (6)

#### MM:

It is a method-of-moment estimator proposed by Huang *et al.* (2014), which is a function of higher-order vector coefficients (Δ). Those coefficients are computed independently for each locus based on the similarity between genotypes. This similarity is formed by two probabilities: i) the probability of observing the genotypes by chance (*i.e.*, when the relatedness is 0), b) the probability of observing the genotypes not by chance.

#### ML:

It is a maximum-likelihood estimator proposed by Huang *et al.* (2015), and it is also a function of Δ. Those coefficients are found by independently maximizing the sum of the log-likelihood of all loci given the search in the parameter space [0; 1]. The likelihood of a locus is $l=Pr\left(S|\Delta \right)$, where *S* is the probability to observe each identity-by-state configuration conditioned to a particular identical-by-descent mode.

#### VR:

It is an extension of VanRaden (2008) presented in Ashraf *et al.* (2016) where ${\hat{r}}_{VR}$ is equal to the off-diagonal elements of the genomic relationship matrix ($A_{VR}$):

$$A_{VR}=\frac{ZZ^{T}}{\sum_{l=1}^{L}s_{l}^{2}}$$ (7)

where *Z* is a matrix of markers *M* centered toward zero; *M* has individuals on rows and *L* loci on columns; each genotype is represented by the number of copies of the referred allele (*e.g.*, 0, 1, 2, …, 2v) and $s_{l}^{2}$ is the variance of locus *l*.

#### PD:

It is an extension of Yang *et al.* (2010) and presented in Slater *et al.* (2016) as “pseudo-diploid” (PD) model:

$${\hat{r}}_{PD}=\frac{1}{L}\overset{L}{\underset{l=1}{\sum }}\frac{\left(m_{xl}-2p_{l}\right)\left(m_{yl}-2p_{l}\right)}{2p_{l}\left(1-p_{l}\right)}$$ (8)

where $p_{l}$ is the frequency of the reference allele in the locus *l*, $m_{xl}$ and $m_{yl}$ are the genotype for locus *l* for individuals *x* and *y*. In this “pseudo-diploid” method, all the heterozygous genotypes are coded as 1 and the homozygous are either 0 or 2.

#### FA:

It is presented in Slater *et al.* (2016) as the “full-autopolyploid” (FA) model:

$${\hat{r}}_{FA}=\frac{1}{\left(2v+1\right)5L}\overset{\left(2v+1\right)5L}{\underset{l\prime =1}{\sum }}\frac{\left(m_{xl\prime }-p_{l\prime }\right)\left(m_{yl\prime }-p_{l\prime }\right)}{p_{l\prime }\left(1-p_{l\prime }\right)}$$ (9)

where $p_{l\prime }$ is the frequency of the individuals carrying the given locus genotype. It considers *L* loci and each locus can have 2v+1 genotypes coded with 0 or 1. This marker parameterization is also known as ”general” model (Rosyara *et al.* 2016).

We implemented the method based on the pedigree and the VR, PD, and FA approaches in the R (R Core Team 2020) package AGHmatrix V2.0 (Amadeu *et al.* 2016) available at https://CRAN.R-project.org/package=AGHmatrix repository. The other approaches (RI, LO, WE, MM, and ML) are detailed and implemented in PolyRelatedness V1.6 software (Huang *et al.* 2016).

### Comparison of methods

In order to compare the estimators, Pearson’s correlation (*ρ*) between ${\hat{r}}_{method}$ and ${\hat{r}}_{obs}$ was computed for the genotypes derived from the genealogy of high- and low-inbreeding genotypes for each method. ${\hat{r}}_{obs}$ is measuring the true average relationship that was simulated, and ${\hat{r}}_{method}$ is a estimator of this true relationship. Additionally, Lin’s concordance correlation coefficient (Lawrence and Lin 1989) and the root mean square error between $\hat{r}$’s were also computed. To compare the methods regarding estimation of specific relationships, we used interval of confidence (IC). The IC, as defined in Huang *et al.* (2016), is the percentage of ${\hat{r}}_{method}$ that relies on the interval ${\hat{r}}_{obs}\pm 0.05$.

### Data availability

The scripts to simulate and reproduce all the analysis necessary for confirming the conclusions of the article are located at https://www.github.com/rramadeu/PolyMolRel_SupMat as well as a tutorial to perform the scripts. Supplemental material available at figshare: https://doi.org/10.25387/g3.12808349.

## Results and Discussion

**Figure 2:**
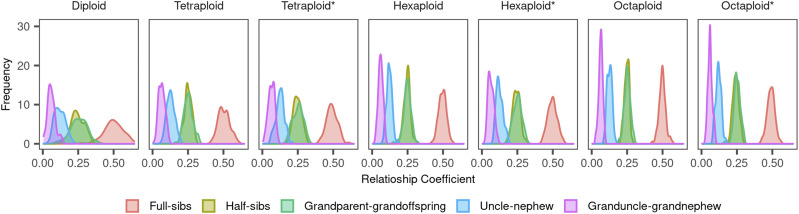
Density plot of Mendelian sampling variation on 100 simulated populations for each ploidy, meiotic event, and relationship. Genotypes simulated considering 10 chromosomes of 100 cM with locus every 0.1 cM summing 10,000 loci in the genome using different ploidy levels and meiotic pairing. *, meiosis with multivalent pairing which allows multivalent formation and double-reduction, without *, meiosis with only bivalent formation.

We studied the sampling variance of the observed relatedness (Figure 2) and the effect of different relatedness estimators using simulated populations for a combination of ploidy, meiotic pairing, inbreeding, number of loci, and number of alleles per loci. We described and compared the pattern of the different tested methods, and, then, recommended the best ones. Statistics used to compare methodologies are presented in (Supplementary Data S1).

Mendelian sampling variance shrinks as the ploidy level increases and enlarges with multivalent pairing (Figure 2 and Supplementary Table S1). Neither ploidy nor type of pairing seems to affect the average relatedness (which is close to the expected value based on identical-by-descent). Both phenomena, maintenance of the mean relatedness and changing in variance, are similar across relationships and reflects the expected segregation pattern. This follows the expected results. To illustrate it, consider a small example of one locus with bivalent meiosis in a $F_{2}$ population. The autotetraploid segregation would be 1 AAAA: 4 ABBB: 6 AABB: 4 ABBB: 1 BBBB resulting in a homozygous proportion of 2/16. On the other hand, in the diploid case, the homozygous proportion would be 2/4 (assuming 1:2:1 segregation in a $F_{2}$). The relative higher proportion of individuals in the tails of the distribution results in a higher variance of diploid full-sibs compared with autopolyploid full-sibs (and also for the other relationships). On the other hand, if there is double-reduction (in the multivalent scenarios), there would be a higher chance of obtaining homozygous gametes, and, therefore, the sampling variance increases. Hill and Weir (2011) presented analytical equations to compute Mendelian sampling variance for non-inbred diploid individuals in a study based on the autosomal human genome (2n = 2x = 44). The diploid results presented herein are similar to the standard deviations found by them. To our knowledge, there is no study about Mendelian sampling variance in autopolyploids. Our results present a first evidence of how ploidy level and pairing would affect the actual relationship between individuals considering linkage. This Mendelian sampling variance behavior is associated to buffering effect of polysomic inheritance and can shed lights on the genetic basis of buffering effect. By definition, buffering effect is related to the masking of beneficial alleles and results in retarded allele fixation in autopolyploids and, consequently, reduction of the genetic variance of quantitative traits for autopolyploids (Soltis *et al.* 2014).

**Figure 3:**
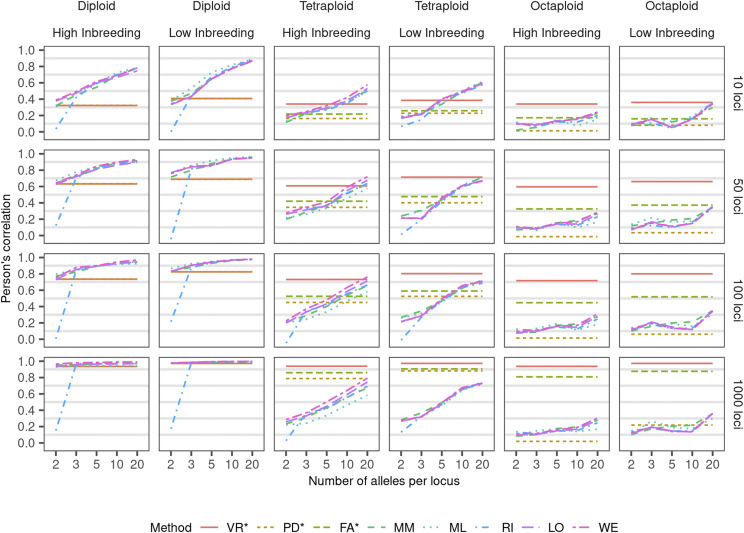
Correlation between observed and estimated $\hat{r}$ based on 100 replicates across numbers of loci and alleles on different ploidies based on simulated genotypes of two pedigrees with high and low inbreeding assuming uniform distribution of ancestral alleles. Methods: VR (extended Van Raden), PD (pseudo-diploid), FA (full-autopolyploid) MM (method-of-moments), ML (maximum-likelihood), RI (extended Ritland), LO (extended Loiselle), and WE (extended Weir). *Biallelic methods (VR, PD, and FA) considered only two alleles scenarios, plotted line extended to better comparison.

**Figure 4:**
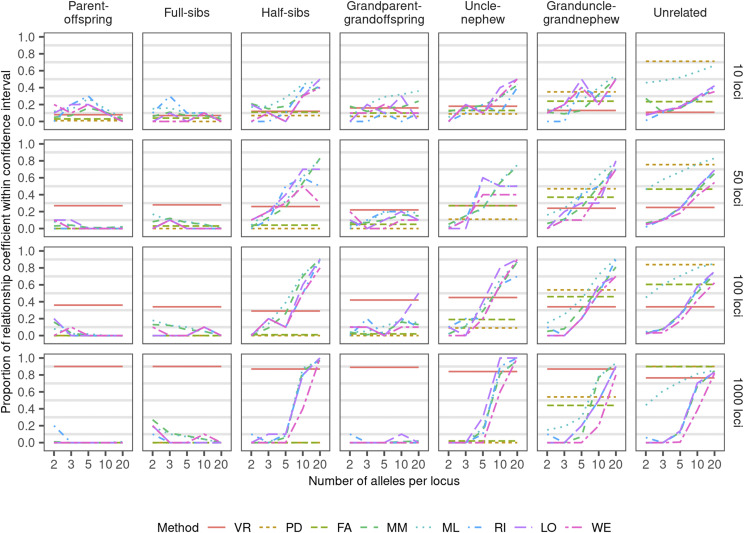
Proportion of relationship coefficient within a confidence interval of ${\hat{r}}_{obs}\pm 0.05$ assuming autotetraploid genotypes with different numbers of loci and alleles based on simulations with uniform distribution of ancestral alleles for each relationship. Methods: VR (extended Van Raden), PD (pseudo-diploid), FA (full-autopolyploid) MM (method-of-moments), ML (maximum-likelihood), RI (extended Ritland), LO (extended Loiselle), and WE (extended Weir). Biallelic methods (VR, PD, and FA) were just used for two alleles. *Biallelic methods (VR, PD, and FA) considered only two alleles scenarios, plotted line extended to better comparison.

The amount of molecular information was not linearly related with the better estimation. For multiallelic methods (MM, ML, RI, LO, or WE), an increase in the number of alleles results in a better estimation of the relatedness (Figure 3). However, for those methods, when the number of loci increases, this trend is only observed in diploids. For our results, the three evaluated metrics (root mean square error, Lin’s concordance correlation coefficient, and Pearson’s correlation (*ρ*) had similar interpretation, therefore, we discuss our results in terms of just one metric, the Pearson’s correlation, which is a widely known metric in the community. Root mean square error and Lin’s concordance correlation coefficient results are in Supplementary Figure S1 and Supplementary Figure S2. In autopolyploids with 100 loci or more (despite the number of alleles), the VR methodology has the highest *ρ*. In diploids and autotetraploids with 50 loci or less, multiallelic methods had higher *ρ* under high number of alleles. The proportion of estimated relatedness coefficient within an interval of confidence of ±0.05 of the observed relatedness (IC) changes depending on the degree of relationship between individuals and the number of alleles and loci considered (Figure 4). For the relationships half-sibs, uncle-nephew, and granduncle-grandnephew, the higher number of loci and alleles, higher the IC across all methods. For the relationships parent-offspring, full-sibs, and grandparent-grandoffspring, only the VR method presented an increase of IC with increasing number of loci. The granduncle-grandnephew and unrelated relationship interpretation needs caution about the IC statistics. PD and FA methods showed the highest ICs, but such estimators are biased toward zero, which is the relatedness between unrelated individuals (Figure 4). In these two methods, almost every genotype falls in the [0:0.05] interval which overlaps the IC interval for unrelated and a high proportion of the granduncle-grandnephew sampling variation (Figure 2 and Supplementary Table S1). Thus, for low ${\hat{r}}_{obs}$, a high IC can be misleading depending upon the estimator bias. Meiotic pairing pattern (bivalent or multivalent) did not affect any of the estimators (Supplementary Figure S3).

Overall, VR and correlation methods performed better than similarity-based methods (MM, ML, RI, LO, and WE, Figure 3). On the correlation methods, VR does not make genetic assumptions (as Hardy-Weinberg Equilibrium or gene independence) and the computation is based on the correlation between the pairwise marker vectors. When hundreds of loci are being used, this method is similar to Wright (1922) for quantitative traits. PD assumes no distinction between heterozygous and bias the results in several scenarios (Supplementary Figure S4). FA, by construction, does not account for dosage and has a bias toward zero (Supplementary Figure S5). On the other hand, MM, ML, RI, LO, and WE are methods based on the similarity index. Similarity measures the relative distance between points within the parametric space [0:1]. After finding such similarity indices, the $\hat{r}$ is computed differently for each method. Therefore, in those, $\hat{r}$ is not computed directly from a correlation coefficient. This difference alongside with model assumptions (as Hardy-Weinberg Equilibrium and no linkage between loci) seems to influence the performance of these methods. Assuming 20 unlinked loci and a natural population, conversely, Meirmans *et al.* (2018) showed high resemblance between simulated and estimated $\hat{r}$ which were not observed here. Additionally, $\hat{r}$ estimated based on multiallelic methods had an odd pattern clustering the estimates into clouds related with the similarity coefficients (Supplementary Figure S5). Since this grouping can bias Pearson’s correlation, we investigated additional statistics as concordance correlation coefficient and root mean square error (Supplementary Data S1), but all statistics showed similar interpretation as *ρ*, which was kept to lead this discussion.

Using the VR method, the low-inbreeding genealogy has on average 0.06 points higher *ρ* than the high-inbreeding genealogy (Supplementary Data S1). Until unrelated ancestors are reached, the high-inbreeding genotype has more generations in the genealogy than the low-inbreeding genotype, and also more inbreeding events (Figure 1). Considering no double-reduction, the expected inbreeding based exclusively on the pedigree records of the low-inbreeding pedigree is 0.0007 and for the high-inbreeding is 0.0354 (47x higher). Such high homozygosity disturbs relatedness estimation and may underestimate it (points shifted to the left on the estimated relatedness of high-inbreeding pedigree in Supplementary Figure S6). Conversely, the inbreeding due to double-reduction given the inheritance pattern (polysomic *vs.* disomic scenarios), does not seem to affect the methods performance in the relatedness estimation.

In autopolyploids, several genomic-assisted selection studies (Li *et al.* 2015; Annicchiarico *et al.* 2015; Slater *et al.* 2016) treat allele dosage in polyploids with no distinction between heterozygous classes (a.k.a. as pseudo-diploid or diploidized model). Despite the allele frequency computation of the estimator, the method PD has such diploid characteristic and can be used to compare the effect of calling dosage. In the autotetraploid population with biallelic loci, as the ratio between alleles decreases, *ρ* statistics value increases for PD method (Supplementary Figure S4 and Supplementary Data S1). In the extreme simulated case 1:9 - where *ρ* presented the highest value - it is expected a higher amount of homozygous and simplex classes. In this specific scenario, considering the data as diploid or polyploid would have the same information. However, this trend of PD with low allele ratio and higher *ρ* is not observed in higher ploidies. Therefore, our results suggest that to consider diploid dosage for autopolyploid analyses results in a lower *ρ*. This noteworthy impacts downstream application as in genomic selection where autotetraploid studies have shown a higher (or at least similar) predictive accuracy of dosage models when compared with diploidized models (de Bem Oliveira *et al.* 2019; Matias *et al.* 2019; de C. Lara *et al.* 2019).

Our results show that it is possible to obtain a consistent estimation with more than 100 biallelic markers under the VR method. While comparing biallelic and multiallelic scenarios for more than 100 loci, we noticed that biallelic VR method performs better for all criteria. Therefore, with the available methods for relatedness estimation, the use of a few hundreds of effective SNP markers results in a more reliable estimation than using hundreds of multiallelic markers (microsatellites or even haplotypes). If the researcher wants to achieve an overall relatedness estimation with high accuracy (ρ≥0.8), it is recommended to use at least 100 effective biallelic markers (Supplementary Data S1). With multiallelic markers, such power is just achieved in some cases with 100 or more markers, each one with 15 or more alleles. With the current available markers (as microsatellites), such allelic numbers might be unrealistic to achieve. This observation might sound counter-intuitive but it is a good result since illustrates that is possible to estimate relatedness with high accuracy using a high-throughput and widely used molecular marker as SNP. It also illustrates the necessity to develop new methodologies that considers multiallelic markers. Noteworthy that our results do not account for the additional noise from the use of precedent methods to infer microsatellite dosage which may cause additional loss of power (Dufresne *et al.* 2014).

The number of markers here recommended to have a good relatedness estimation will vary according of population linkage disequilibrium, effective population size, number of independent chromosome segments ($M_{e}$) (van den Berg *et al.* 2015), and number of effective independent SNP markers in the population (Misztal 2016). For instance, in our simulations, we set the ancestral alleles with no linkage disequilibrium and, therefore, our markers are effective markers - in the simulations with 1,000 loci, $M_{e}=1,000$. $M_{e}$ for a real data set can be computed based on linkage disequilibrium, on the covariance between non-relative relatedness, or on effective population size, and genomic characteristics (Lee *et al.* 2017). Using empirical data, 3,895 polymorphic SNPs were used in the construction of the genomic relationship matrix in autotetraploid potato (Endelman *et al.* 2018). Assuming $M_{e}=1/var\left(A_{VR\prime }\right)$, where $var\left(A_{VR\prime }\right)$ is the covariance between the genomic pairwise relatedness of unrelated individuals (Lee *et al.* 2017), we obtain an $M_{e}=140.3$, lower than the initially 3,895 SNPs, but above the threshold of 100 biallelic markers set in this present study; therefore, it reflects a reliable *r* estimator for the overall relationship. However, this $M_{e}$ is far from the ideal to estimate specific inbred relationships estimation. As we observed, it is necessary almost 1,000 effective markers for IC≥0.8 (Figure 4) which can be unfeasible depending upon the population parameters. If a highly related population is analyzed (as a population of full-sib families with related parents, a common scenario in breeding programs), a high number of molecular markers may not represent a high number of effective markers; thus, even a population with thousands of loci may have low $N_{e}$ and $M_{e}$, which might translate in a $\hat{r}$ estimation with low accuracy.

This study extends to future computations based on haplotypes. They can be derived from long and whole genome sequencing platforms (Lam *et al.* 2012; Rhoads and Au 2015), or through haplotype assembling methods which consider ploidy and SNP markers as implemented in Aguiar and Istrail (2013) and Das and Vikalo (2015), or through probabilistic haplotype reconstruction based on mapping populations as implemented in Zheng *et al.* (2016) and Mollinari and Garcia (2019). All above technologies may be used to gather thousands of multiallelic markers. From this work, we show the necessity to new theoretical and computational developments to compute estimate relatedness for multiallelic markers. Future methods must account for linkage, using information from physical or genetic map position. Moreover, there is the possibility to consider mixed segregation with disomic and polysomic inheritance and to include genotyping errors in future methods. Due to simulation limitations, we fixed a single cytotype for the entire population, however there are recent methods that considers multiple cytotypes and multiallelic dosage information (Huang *et al.* 2020) which needs further investigation of their performance within breeding populations. Also, we did not consider preferential pairing in the simulations. Our results are extended for many autopolyploid crops with low evidence of preferential pairing as potato (Bourke *et al.* 2015), blueberry (Lyrene *et al.* 2003), and alfalfa (Cao *et al.* 2004), however, to extend our results to crops with unknown genomic transmission pattern is not recommended.

## Conclusion

Here, we show that a biallelic method performed usually better than the actual multiallelic methods and, nowadays, an effort to obtain multiallelic markers may be of reduced value to estimate relatedness. In just a few specific scenarios - with highly unbalanced allele frequency - pseudo-diploid methods may be satisfactory. Therefore, methods specifically developed for polyploids must be used. Relatedness estimated with the available multiallelic markers only have a high accuracy with more than 100 loci and many alleles which is currently unrealistic. To infer relatedness with high accuracy in a highly inbred autopolyploid population is harder than in a population with low inbreeding rates or unrelated. Nowadays, to achieve high accuracy in the relatedness estimation in autopolyploids, we recommend more than 100 effective biallelic SNP markers with reliable dosage inference and the extended VR method.
